# Comparison of SensPERT® *Leishmania* rapid test with two other immunochromatographic tests for the diagnosis of canine visceral leishmaniasis in Brazil

**DOI:** 10.14202/vetworld.2024.1307-1310

**Published:** 2024-06-19

**Authors:** Mariana Elisa Pereira, Maria Clara Bianchini Neves, Arleana do Bom Parto Ferreira de Almeida, Valéria Régia Franco Sousa

**Affiliations:** 1 Postgraduate Program in Veterinary Science, Faculty of Veterinary Medicine, Federal University of Mato Grosso, Cuiabá, Mato Grosso, Brazil; 2 Graduate Program in Veterinary Medicine, College of Veterinary Medicine, Federal University of Mato Grosso, Cuiabá, Mato Grosso, Brazil; 3 Faculty of Veterinary Medicine, Federal University of Mato Grosso, Cuiabá, Mato Grosso, Brazil

**Keywords:** immunochromatography, *Leishmania infantum*, reservoir

## Abstract

**Background and Aim:**

In urban environments, dogs serve as the primary reservoir for visceral leishmaniasis (VL). Rapidly diagnosing canine VL through tests enables early treatment and a favorable prognosis. This study aimed to assess the diagnostic performance of the SensPERT® *Leishmania* test kit (Dechra®), Alere® Leishmaniasis Ac test kit, and the rapid test dual path platform (TR-DPP®) Bio-Manguinhos in detecting VL.

**Materials and Methods:**

30 serum samples from reactive VL dogs and 30 serum samples from healthy dogs were employed for assessing the sensitivity and specificity variation between SensPERT® *Leishmania* test kit, Alere® Leishmaniasis Ac test kit, and rapid test dual platform – TR-DPP®.

**Results:**

The SensPERT® *Leishmania* test outperformed Alere® and TR-DPP® in terms of sensitivity, specificity, positive and negative predictive values and demonstrated near-perfect concordance with Alere® and substantial concurrence with TR-DPP®.

**Conclusion:**

The SensPERT® *Leishmania* rapid test proved to be a promising test in the detection of VL in dogs.

## Introduction

Leishmaniasis is caused by *Leishmania* parasites that infect humans and several animal species. Parasites, typically with higher diversity and greater force of infection [1], are crucial in the New and Old World’s tropical and subtropical regions [2]. The estimated incidence of visceral leishmaniasis (VL) is 200,000–400,000 new cases per year, with approximately 90% of them occurring in Bangladesh, Brazil, Ethiopia, India, South Sudan, and Sudan [3]. 95% of all VL cases in the Americas occur in Brazil. All Brazilian regions are endemic to VL [4], *Leishmania infantum* (syn. *Leishmania chagasi*) is the etiological agent of VL in Brazil [5]. Untreated VL results in severe illness and death, primarily in children and immuno-suppressed individuals, manifested with hepatosplenomegaly, weight loss, and anemia [6].

Due to urbanization caused by deforestation, agriculture, and rural exodus, dogs are crucial in disease transmission. In many regions, urban reservoirs primarily harbor the parasite preceding human VL occurrence [7]. Endemic areas pose a significant challenge to diagnosing canine monocytic ehrlichiosis due to the myriad of clinical signs and numerous asymptomatic dogs [8]. Leishmaniasis diagnosis involves parasite identification on Romanowsky-stained slides, parasite culture, laboratory animal inoculation, polymerase chain reaction (PCR), indirect immunofluorescence assay, and enzyme-linked immunosorbent assay (ELISA) [9]. These techniques come with a hefty price tag, requiring specialized teams, costly equipment, and considerable time. In Brazil, the Ministry of Health recommends the rapid test dual path platform (TR-DPP® canine visceral leishmaniasis Bio-Manguinhos/FIOCRUZ, Rio de Janeiro, RJ, Brazil), which uses antigen rK28 (a chimaera combining antigens K9, K26, and K39) of *L. infantum* [10] as a screening test and enzyme immune assay for diagnosis of canine visceral leishmaniasis (EIE-LVC® Bio-Manguinhos/FIOCRUZ) as a confirmatory test [11].

Other commercially available rapid immunochromatographic tests as screening tests provide rapid diagnosis without requiring large infrastructure or a specialized laboratory diagnostic team. TR-DPP® canine visceral leishmaniasis (CVL) has 89% sensitivity and 70% specificity when compared to parasitological culture, immunohistochemistry, and histopathology [10]. Alere® Leishmaniasis Ac Test (Alere, São Paulo, SP, Brazil), which uses recombinant protein rK28 as the antigen, has 85% sensitivity in comparison to serological and parasitological tests [12]. According to the manufacturer, SNAP® *Leishmania* IDEXX (IDEXX, Markham, Canada) has 99.2% specificity and 96.3% sensitivity [13], this last test showed greater agreement with TR-DPP® CVL than with Alere®. Another immunochromatographic test is SensPERT® *Leishmania*, and despite having similar results to ELISA when dogs were analyzed in the province of Istanbul, Turkey [14], it has not yet been compared with some of the rapid tests used in Brazil. Furthermore, in the control program in Brazil, they use TR-DPP® as an official screening test in seroepidemiological surveys; however, it is not enough to meet the demand of private institutions [10, 13].

Therefore, this study aimed to determine the sensitivity, specificity, and agreement of SensPERT® *Leishmania* test (Dechra®, Londrina, Paraná, PR, BR) and to compare it with other VL immunochromatographic diagnosis tests available in Brazil (Alere® and TR-DPP®).

## Materials and Methods

### Ethical approval

This study was approved by the Ethics Committee on the Use of Animals of the Federal University of Mato Grosso (approval number: 23108.096261/2020-76).

### Study period and location

From December 2020 to February 2022, samples were analyzed from dogs attended at the Small Animal Medical Clinic of the Veterinary Hospital of the Federal University of Mato Grosso (HOVET-UFMT), Cuiabá, Mato Grosso, Brazil (15° 35’ 56” S and 56° 06’ 01” W).

### Samples collection

The study analyzed serum samples from 30 dogs known to be reactive for VL, by the indirect immunofluorescence test (Bio-Manguinhos®, FIOCRUZ, Rio de Janeiro, Brazil) and with DNA amplification using conventional PCR. In addition, serum samples from 30 healthy dogs (blood donors) that were non-reactive for VL and without DNA amplification were collected from the serum bank of the Leishmaniasis Laboratory at HOVET-UFMT, Cuiabá campus. All PCR-positive dogs were symptomatic and presented with lymphadenopathy, skin lesions, onychogryphosis, ophthalmopathy, or weight loss and/or muscle mass loss [15].

### Tests and procedures

The SensPERT® *Leishmania* test (Dechra®, Paraná, Brazil) was compared with TR-DPP® (Bio-Manguinhos®, FIOCRUZ)) and Alere® Leishmaniasis Ac test (Alere®, São Paulo, Brazil), two rapid qualitative tests used for the canine serum samples. All rapid tests were performed according to the manufacturer’s instructions and interpreted by three researchers. The SensPERT® *Leishmania* rapid test was observed at 5 and 10 min intervals.

Anti-*Leishmania* antibody titers were determined using the indirect immunofluorescence antibody test (IFAT, Bio-Manguinhos®, FIOCRUZ) according to the manufacturer’s recommendations. Samples were considered positive when a reaction was observed at a serum dilution of 1:40 or higher [16].

DNA was extracted from leukocyte-covered samples for molecular detection by PCR using the phenol/chloroform/isoamyl alcohol method [17]. The extraction product was eluted in Milli-Q water and stored at −80°C until use. The primers 150 (sense) 5’-GGCCCACTATATTACACCAACCCC-3’ and 152 (antisense) 5’-GGGGTAGGGGCGTTCTGCGAA-3’ were used to amplify a fragment of 120 base pairs of the conserved region of the kDNA minicircle of all *Leishmania* species [18]. Subsequently, primers RV1 (sense) 5’-CTTTTCTGGTCCCGCGGGTAGG-’3 and RV2 (antisense) 5’-CCACCTGGCCTATTTTACACCA-’3 were used to identify the parasite at the complex level, amplifying a fragment of 145 base pairs from the conserved region of the kDNA minicircle of the *Leishmania donovani* complex [19]. The reactions and conditions of the two PCR protocols were modified according to the study by Almeida *et al*. [20].

### Statistical analysis

The results were arranged in a 2 × 2 contingency table to determine sensitivity, specificity, accuracy, positive predictive value (PPV), negative predictive value (NPV), and Kappa agreement between the SensPERT® *Leishmania* test and other analyzed tests. The Kappa agreement between the tests was evaluated as follows: no agreement, <0; mild agreement, 0–0.20; reasonable agreement, 0.21–0.40; moderate agreement, 0.41–0.60; substantial agreement, 0.61–0.80; and near-perfect agreement, 0.81–1 [21].

## Results

Rapid tests with samples from both reactive and non-reactive dogs for CVL were valid. In all SensPERT® *Leishmania*, TR-DPP®, and Alere® rapid tests, the control line was observable. The SensPERT® test revealed substantial agreement with TR-DPP® and almost perfect agreement with the Alere® *Leishmania* Ac test. The SensPERT® *Leishmania* test’s sensitivity, specificity, PPV, NPV, and Kappa coefficient values are presented in Table-1. At 5 and 10 min during the SensPERT® *Leishmania* test analysis, the positive line appeared with no difference in the outcome. At the 10 min mark, the positive lines appeared darker.

**Table-1:** TR-DPP (Bio-Manguinhos®) and Ac test (Alere®) sensibility, specificity, PPV, NPV, and Kappa coefficient compared with the SensPERT® *Leishmania* rapid test values.

Test	SensPERT® *Leishmania*		Sensibility (%)	Specificity (%)	PPV (%)	NPV (%)	KC	p-value

	Positive	Negative						
TR-DPP®								
Positive	28	5	96.55	83.87	84.84	96.29	0.80	<0.001
Negative	1	26
Alere®								
Positive	27	0	93.10	100	100	93.94	0.93	<0.001
Negative	2	31						

PPV=Positive predictive value, NPV=Negative predictive value, TR-DPP=Rapid test dual path platform

The PCR and IFAT tests reacted positively to samples from the dogs diagnosed with LVC. In the IFAT test, the titer readings varied as follows: One dog at 1:40, five dogs at 1:80, six dogs at 1:160, nine dogs at 1:320, and nine dogs at 1:640. The SensPERT® *Leishmania* test showed no reaction for samples from the two dogs with titers of 40 and 80. None of the healthy dogs presented *L. infantum* DNA amplification, none reacted positively in IFAT (≥1:40), and only one tested positive in the SensPERT® *Leishmania* assay.

## Discussion

Rapid diagnosis of *L. infantum* in dogs in urban environments is essential for both regulatory control and the dog’s health, and the SensPERT® *Leishmania* test demonstrates near-perfect agreement with the *Leishmania* Ac test (Alere®) for this purpose. This field test provides another opportunity for identifying potential cases for prompt decision-making. However, given the wide variety of clinical signs, non-specific histopathological changes, and the absence of a 100% specific and sensitive laboratory test [16], a combination of serological qualitative or quantitative tests, as well as molecular analyses, increases the probability of detection in dogs with *L. infantum* infection [22].

Among the dogs with CVL confirmed by IFAT and PCR, two dogs had false-negative results, while one healthy dog had false-positive results in the SensPERT® *Leishmania* test. False-negative dogs showed low antibody titers in the IFAT. False-positive and false-negative results may lead to the elimination of uninfected dogs and facilitate the spread of the disease, respectively [23], demonstrating the importance of using reliable techniques and laboratory tests.

The performance of serological tests varies according to the clinical course, the immunological response of the host, and the type of antigen or immunoreagent used [24]. The SensPERT® test with K26 and K39 recombinant proteins demonstrated superior sensitivity, specificity, PPV, and NPV compared to the TR-DPP® and *Leishmania* Ac test using rK9, rK26, and rK39 antigens of *L. infantum*, resulting in rK28 [10, 13]. These three immunochromatographic tests have the proteins K26 and K39 in common, which are kinesin-conserved antigens, to evaluate the sensitivity and specificity of the detection of antibodies against VL in humans and dogs [25].

The agreement levels between SensPERT® and TR-DPP®, as well as SensPERT® and Alere® *Leishmania* rapid tests, for the diagnosis of CVL were substantial and almost perfect, respectively. The SensPERT® *Leishmania* test is a suitable option for diagnosing *L. infantum* antibodies in dogs, requiring minimal equipment and ease of use for veterinary professionals. The SensPERT® *Leishmania* rapid test is recommended for use in conjunction with other diagnostic methods for CVL [10, 12].

A non-reactive dog in the IFAT from the healthy dog group had false-positive results in the SensPERT® *Leishmania* rapid test, highlighting the need for additional studies, including in animals that tested positive for other pathogens, to verify the occurrence of cross-reactions [26]. The influence of clinical scores on the performance of SensPERT® *Leishmania* and TR-DPP® tests is an intriguing question [10]. The TR-DPP® rapid test has greater sensitivity in symptomatic dogs than in asymptomatic animals [24]. This study was unable to confirm the preliminary finding due to the presence of symptoms in all detected dogs and a small sample size.

## Conclusion

The SensPERT® *Leishmania* rapid test was found to effectively detect canine VL. Further research with larger groups is required to substantiate these results. It is essential to include asymptomatic dogs when assessing test sensitivity and specificity in this population.

## Authors’ Contributions

MEP: Conceptualization, data curation, formal analysis, methodology, software, and writing – original draft. MCBN: Data curation, formal analysis, and writing – original draft. ABPFA: Data curation and supervision. VRFS: Conceptualization, data curation, formal analysis, investigation, methodology, project administration, resources, supervision, validation, and writing – review and editing. All authors have read, reviewed, and agreed to the published version of the manuscript.
